# Rapid and coarse face detection: With a lack of evidence for a nasal-temporal asymmetry

**DOI:** 10.3758/s13414-019-01877-3

**Published:** 2020-01-06

**Authors:** Laura Cabral, Bobby Stojanoski, Rhodri Cusack

**Affiliations:** 1 Brain and Mind Institute, Western University, London, ON N6A 5B7 Canada; 2 Trinity College Institute of Neuroscience, Trinity College Dublin, Dublin, Ireland

**Keywords:** Rapid processing, Face detection, Subcortical, Retinocollicular pathway, Nasal temporal asymmetry

## Abstract

Humans have structures dedicated to the processing of faces, which include cortical components (e.g., areas in occipital and temporal lobes) and subcortical components (e.g., superior colliculus and amygdala). Although faces are processed more quickly than stimuli from other categories, there is a lack of consensus regarding whether subcortical structures are responsible for rapid face processing. In order to probe this, we exploited the asymmetry in the strength of projections to subcortical structures between the nasal and temporal hemiretina. Participants detected faces from unrecognizable control stimuli and performed the same task for houses. In Experiments 1 and 3, at the fastest reaction times, participants detected faces more accurately than houses. However, there was no benefit of presenting to the subcortical pathway. In Experiment 2, we probed the coarseness of the rapid pathway, making the foil stimuli more similar to faces and houses. This eliminated the rapid detection advantage, suggesting that rapid face processing is limited to coarse representations. In Experiment 4, we sought to determine whether the natural difference between spatial frequencies of faces and houses were driving the effects seen in Experiments 1 and 3. We spatially filtered the faces and houses so that they were matched. Better rapid detection was again found for faces relative to houses, but we found no benefit of preferentially presenting to the subcortical pathway. Taken together, the results of our experiments suggest a coarse rapid detection mechanism, which was not dependent on spatial frequency, with no advantage for presenting preferentially to subcortical structures.

**Electronic supplementary material:**

The online version of this article (10.3758/s13414-019-01877-3) contains supplementary material, which is available to authorized users.

## Introduction

Animals as diverse as fish, birds, and sheep can recognize the faces of their conspecifics (Leopold & Rhodes, 2010). In humans there has evolved a network of structures responsible for face processing that facilitates face detection, orientating, and identification (Haxby, Hoffman, & Gobbini, 2000; Mende-Siedlecki & Verosky, 2013; Tong, Nakayama, Moscovitch, Weinrib, & Kanwisher, 2000). This comprises subcortical components, including the superior colliculus and amygdala (Mende-Siedlecki & Verosky, 2013; Vuilleumier, Armony, Driver, & Dolan, 2003), and cortical components in the occipital and temporal lobes (Kanwisher, Mcdermott, & Chun, 1997; Kanwisher & Yovel, 2006; Pitcher, Dilks, Saxe, Triantafyllou, & Kanwisher, 2011). These specialized processing mechanisms allow faces to be detected more quickly than objects (Crouzet, Kirchner, & Thorpe, 2010) and result in faces being the first category to be detected in visual search tasks (Fletcher-Watson, Findlay, Leekam, & Benson, 2008). Detecting faces quickly is thought to be evolutionarily advantageous for both survival and social interaction, from the savannahs of Africa to the office party.

The subcortical route via the retinocollicular pathway to the amygdala is often thought to facilitate “quick and dirty” face detection (Johnson, 2005). It comprises projections from the retina to the superior colliculus, which in turn project to the pulvinar nucleus on the way to the amygdala (Benevento & Standage, 1983; Jones & Burton, 1976; Rafal et al., 2015; Tamietto, Pullens, De Gelder, Weiskrantz, & Goebel, 2012) Evidence that the retinocollicular pathway can process faces comes from blindsight patients, who after extensive damage to the visual cortex are still able to detect the emotional content of faces, although they cannot recognize their identity (Tamietto and de Gelder, 2010). Similar behavior is found in healthy controls following transracial magnetic stimulation to the visual cortex; when TMS prevents participants from seeing stimuli, they are still able to recognize the emotional content of the face (Jolij & Lamme, 2005). Furthermore, structures in the retinocollicular pathway are activated by the viewing of neutral and emotional faces, as shown with functional magnetic resonance imaging (fMRI) (Mende-Siedlecki & Verosky, 2013). Functional magnetic resonance imaging has also found that this pathway has a preference for crude, low-spatial frequency information, with greater activation to faces filtered to emphasize low spatial frequencies than high spatial frequencies (Vuilleumier et al., 2003).

Intracranial recordings in epilepsy patients have found that the retinocollicular pathway is fast, with neural firing in the amygdala as quickly as 100–250 ms after the presentation of an emotional face (Sato et al., 2013). Recent intracranial recording from Méndez-Bértolo et al. (2016) has found even faster processing for fearful faces, with firing in the amygdala recorded 74 ms after stimulus onset. Magnetoencephalography (MEG) data suggests even faster processing with responses to emotional faces detected in just 40 ms (Luo et al., 2010). Supporting this hypothesis, Garvert, Friston, Dolan, and Garrido (2014) used dynamic causal modeling of MEG data to conclude that a model with a subcortical component, containing the pulvinar nucleus and the amygdala, more accurately modeled rapid face processing than a model with a singular cortical process.

It has been proposed that these putative fast face detection mechanisms are not limited to subcortical structures, as there is also evidence of rapid mechanisms within cortical areas, such as the inferior occipital gyrus (Pitcher, Walsh, Yovel, & Aviv, 2007; Sadeh, Podlipsky, Zhdanov, & Yovel, 2010). Specifically, an initial feed-forward wave of firing through the cortex could allow for rapid, coarse processing (Cauchoix & Crouzet, 2013; Serre, Oliva, & Poggio, 2007; Vanrullen & Koch, 2001). Electroencephalography (EEG) data from the visual cortex can identify responses just 56 ms after stimulus onset (Foxe & Simpson, 2002), and intracranial recordings in epilepsy patients found that the category of image participants were viewing could be decoded from the first 100 ms of response in visual cortex (Liu, Agam, Madsen, & Kreiman, 2009). MEG data suggest occipitotemporal responses to faces in just 100 ms (Liu, Harris, & Kanwisher, 2002). Barragan-Jason, Cauchoix, and Barbeau (2015) have proposed that even the identification of familiar faces has an initial rapid phase, occurring at 140 ms, that depends on coarse visual information, and behavioral responses to familiar faces can be detected in just 180 ms (Visconti di Oleggio Castello & Gobbini, 2015). To formalize how the cortex could rapidly detect complex visual objects such as faces in real-world scenes, Thorpe and colleagues (Delorme & Thorpe, 2001; VanRullen, Guyonneau, & Thorpe, 2005) proposed a spike-based model of rapid processing. These models have been supported by recordings from V1 in the macaque and cat (Celebrini, Thorpe, Trotter, & Imbert, 1993; Konig, Engel, Roelfsema, & Singer, 1995; VanRullen et al., 2005).

In summary, although not without its critics, many authors have argued for both subcortical and cortical mechanisms for rapid visual processing of faces. Which one, therefore, dominates rapid face detection in healthy participants? One way to address whether rapid face perception is driven by subcortical structures is to target the retinocollicular pathway to the amygdala. Presenting stimuli exclusively to the nasal hemiretina preferentially targets the retinocollicular pathway, as the nasal hemiretina contains more fibers projecting to the superior colliculus. Initial evidence for this asymmetry came from tree shrews, cats, and macaques (Conley, Lachica, & Casagrande, 1985; Harrison, 2015; Perry & Cowey, 1985; Pollack & Hickey, 1979; Sterling, 1973). fMRI evidence in humans has demonstrated that the superior colliculus displays a temporal nasal asymmetry that is not found for the LGN or V1 (Sylvester, Josephs, Driver, & Rees, 2007). Additionally, behavioral studies have demonstrated that a nasal-temporal asymmetry is reflective of input to the superior colliculus. For example, making stimuli only visible to the S cones, which do not provide input to the superior colliculus, eliminates the benefit of presenting to the nasal hemiretina (Bertini, Leo, & Làdavas, 2008).

Our goals in this study were to establish a paradigm for behaviorally quantifying rapid face detection, and to determine whether presenting preferentially to the retinocollicular pathway resulted in improved rapid face detection. Participants were asked to detect faces from amongst unrecognizable control stimuli that were matched to have the same low-level visual features, as quantified with a model of the early visual system (Stojanoski & Cusack, 2014). To determine whether any rapid detection mechanism was specific to faces, we also tested a control condition, requiring detection of another class of visual object, houses.

## Experiment 1

### Methods

To probe rapid face processing, in two blocks participants performed a face-detection task in which they pressed a button as quickly as possible for intact faces, but not for scrambled foil stimuli. In two additional blocks, they were asked to detect houses in a similar manner. In each block, stimuli were presented monocularly, by asking participants to wear an eye patch. This allowed us to target stimuli exclusively to either the nasal or temporal hemiretina. In the right eye, presenting stimuli to the right of fixation targets the nasal hemiretina, while presenting to the left of fixation targets the temporal hemiretina. The opposite is true in the left eye. Within each block, stimuli were randomized across the nasal and the temporal hemiretinas.

#### Participants

Twenty-four individuals (12 males, 12 females, age range 18–21 years) were given course credit for participation in Experiment 1. The non-medical ethics board at the University of Western Ontario reviewed and approved the experimental protocol. All participants provided informed consent, reported normal or corrected-to-normal vision, and were right-handed.

#### Stimuli

Twenty-four face photographs from an online database (http://wiki.cnbc.cmu.edu/Face_Place) and 24 house stimuli, created by Martin, McLean, O’Neil, and Köhler (2013), were used in the study. As the house stimuli had a blurred edge, a custom MATLAB (Mathworks, Natick, MA, USA) script added a blurred edge to the face stimuli, so to appear similar by eye. As the house stimuli were grayscale, face stimuli were also altered to be grayscale.

**Fig. 1:**
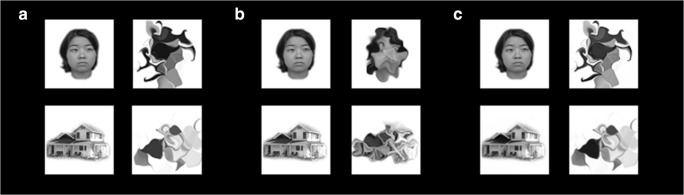
Exemplar stimuli from the four experiments. (**A**) Stimuli used in Experiments 1 and 3. Foil stimuli are unrecognizable versions of faces and houses. (**B**) In Experiment 2, the foil stimuli were more similar to the faces and houses. (**C**) In Experiment 4, the stimuli were adjusted for differences in spatial frequency between categories

All stimuli were centered in a rectangular area of 4.9° × 4.9° of visual angle. The fixation cross was .5° × .5°. A white background was used throughout the experiment. In all experiments, participants viewed the stimuli in a room with the lights on. To generate the control stimuli, faces and houses were diffeomorphically warped using the procedure described by Stojanoski and Cusack (2014). Foils were unrecognizable as determined by the behavioral ratings in Stojanoski and Cusack (2014) (image 38 on the diffeomorphic continuum). A depiction of the stimuli used in Experiment 1 can be found in Fig. 1A. Further details about the stimuli can be found in Supplementary Fig. 1.

#### Procedure

Stimuli were presented on a laptop screen using MATLAB and Psychtoolbox. Participants wore an eye patch to ensure monocular presentation, placed their heads on a chin rest, and were instructed to maintain fixation. The center of the screen was directly ahead of the nose. In each experimental block, a black fixation cross was offset by 3.2 cm to the left or right from center in order to put it directly in front of the unpatched eye. This distance was chosen using the mean interpupillary distance scores from the 1988 Anthropometric Army Survey.

In Experiment 1, participants completed two blocks with their left eye unpatched, one that contained only face targets, the other containing house targets, and two similar blocks with their right eye unpatched. Block order was counterbalanced across participants.

**Fig. 2:**
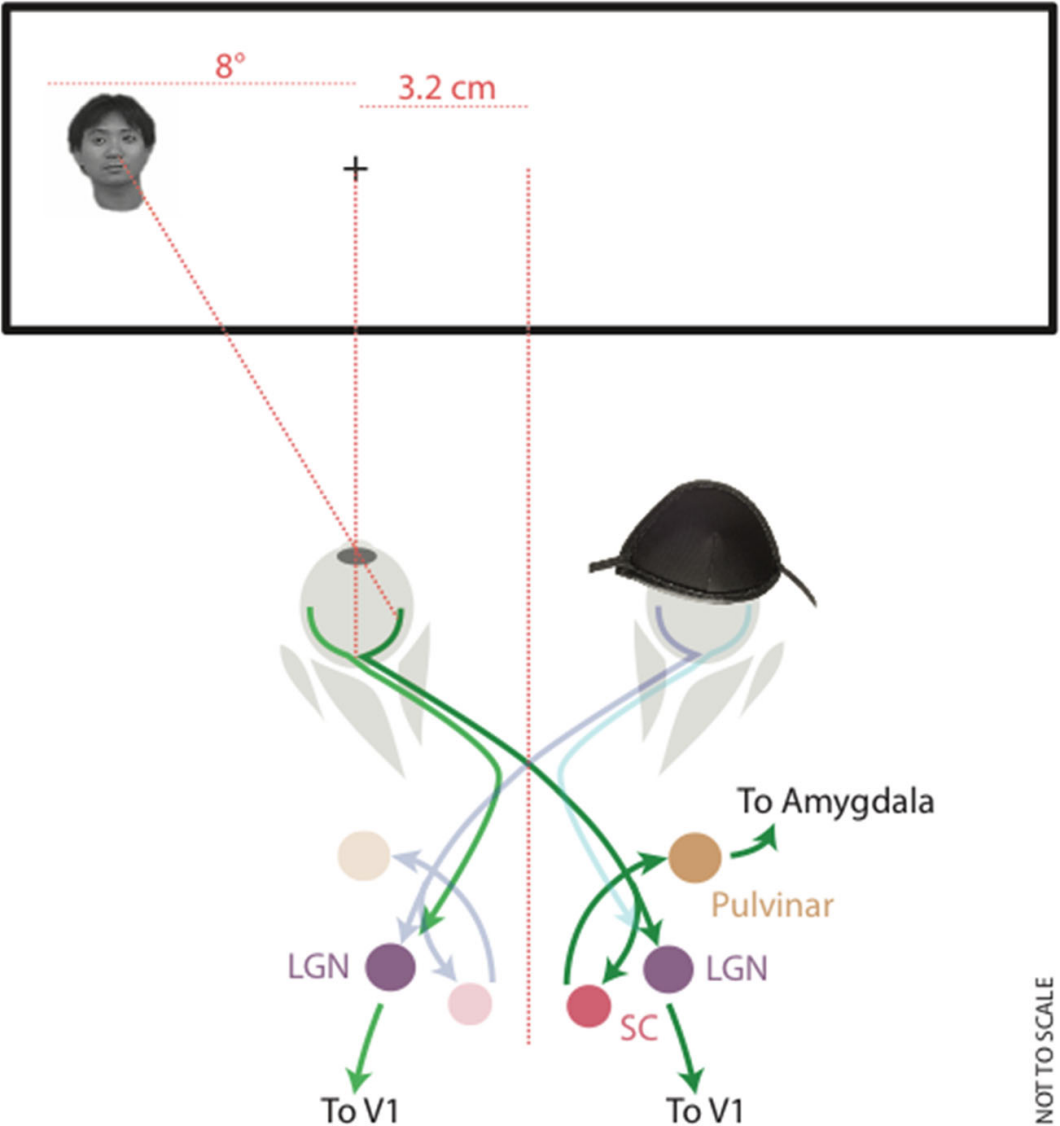
Schematic illustrating the experimental configuration. Participants wore an eye patch to ensure monocular presentation and to allow the stimuli to be presented exclusively to the nasal or temporal hemiretina. The retinocollicular pathway is depicted with projections from the nasal hemiretina to the superior colliculus, through the pulvinar nucleus, terminating in the amygdala. Weaker projections from the temporal hemiretina to the superior colliculus are not shown

In each block, participants were presented with 96 trials comprising two repetitions of 24 target stimuli and their 24 warped counterparts. One repetition was presented to the nasal hemiretina, while the other was presented to the temporal hemiretina. To present to the nasal and temporal visual hemiretina, the stimuli were offset horizontally so that the outer edge of their rectangular bounds was 8° from the center of fixation. Stimuli were presented for duration of 122 ms, with an intertrial interval of 2,505 ms. Participants were instructed to perform a simple detection task, pressing a key a quickly as possible when they saw an intact face (in the face blocks) or an intact house (in the house blocks). For a schematic representation of the experimental configuration, please see Fig. 2.

#### Analysis

In order to quantify rapid processing, we used an analysis strategy similar to Kirchner and Thorpe's (2006) and calculated accuracy for the fastest 10% of responses. All reaction times (RTs) are relative to stimulus onset. A fast detection mechanism would be expected to improve accuracy on these rapid trials by providing more accurate information to decision and action areas sooner after stimulus onset. The RT threshold for the fastest 10% of trials was calculated for each participant individually, in order to account for individual differences in overall reaction time. We also expected that faces would be detected more quickly overall. If this is the case, to ensure that the overall difference in reaction time between the faces and houses did not drive the results, we adopted a conservative analysis strategy and determined the face and house reaction thresholds separately. Thus, the fastest 10% of face trials were expected to be even faster than the fastest 10% of house trials.

To determine the contribution of the retinocollicular pathway, we examined whether presenting the stimuli to the nasal or the temporal hemiretina modulated performance. As the nasal hemiretina has more connections to the superior colliculus and thus the retinocollicular pathway, we would expect to see faces more accurately detected than houses when the stimuli are presented to the nasal hemiretina.

### Results

**Fig. 3:**
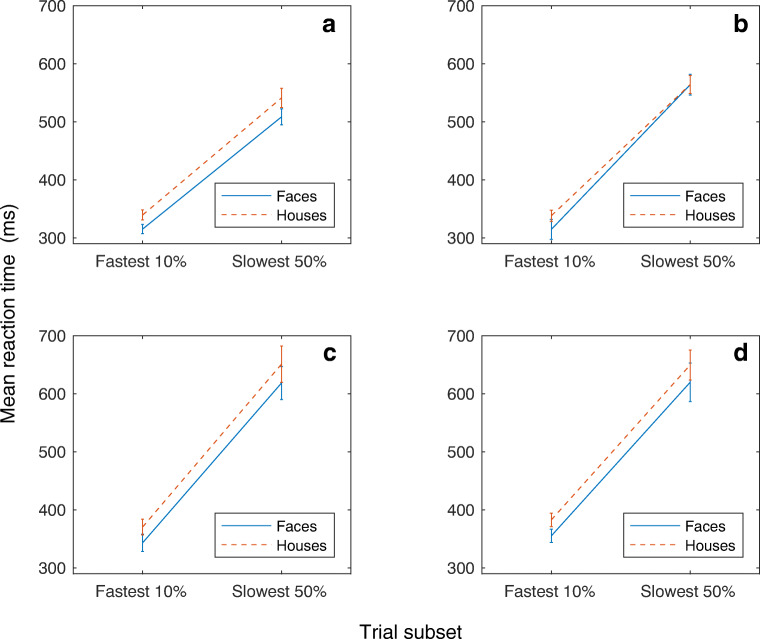
Mean reaction times for the fastest 10% and slowest 50% of trials. (**A**) In Experiment 1, foil stimuli were unrecognizable versions of faces and houses. (**B**) In Experiment 2, faces and houses were more similar to foil stimuli. (**C**) In Experiment 3, stimuli were the same as in Experiment 1. (**D**) In Experiment 4, spatial frequency of the faces and houses were matched. Error bars represent ± 1 standard error

Two participants were excluded for failing to follow the task instructions. Across the remaining participants, mean RTs for both the fastest 10% and the slowest 50% of trials are shown in Fig. 3A. These reaction times include correct responses and false alarms, as both contributed to subsequent accuracy metrics.

**Fig. 4:**
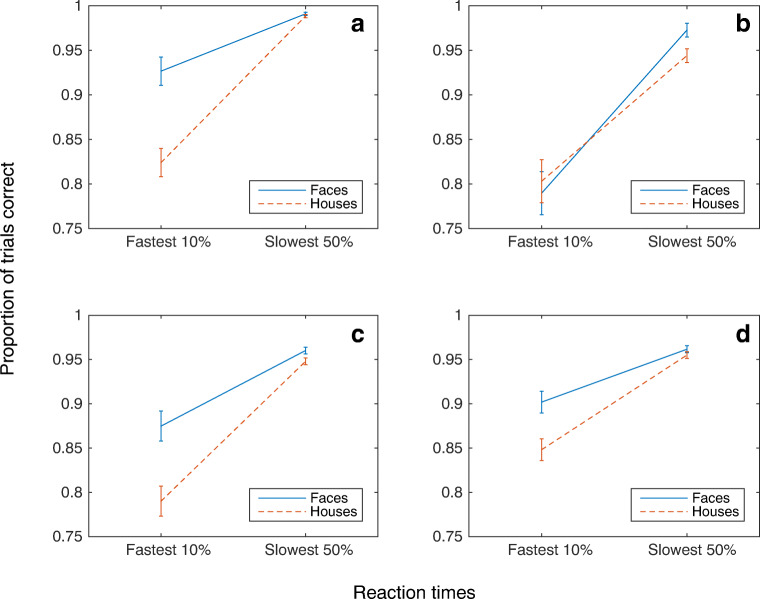
Proportion of trials correct for the fastest 10% and slowest 50% of reaction times. (**A**) In Experiment 1, faces were detected significantly more accurately than houses at the fastest reaction times. (**B**) In Experiment 2, faces and houses were detected with similar accuracy. (**C**) Experiment 3 replicated the results of Experiment 1. (**D**) In Experiment 4, faces were detected significantly more accurately than houses in the fastest 10% of reaction times. In all experiments, error bars represent ± the standard error

**Fig. 5:**
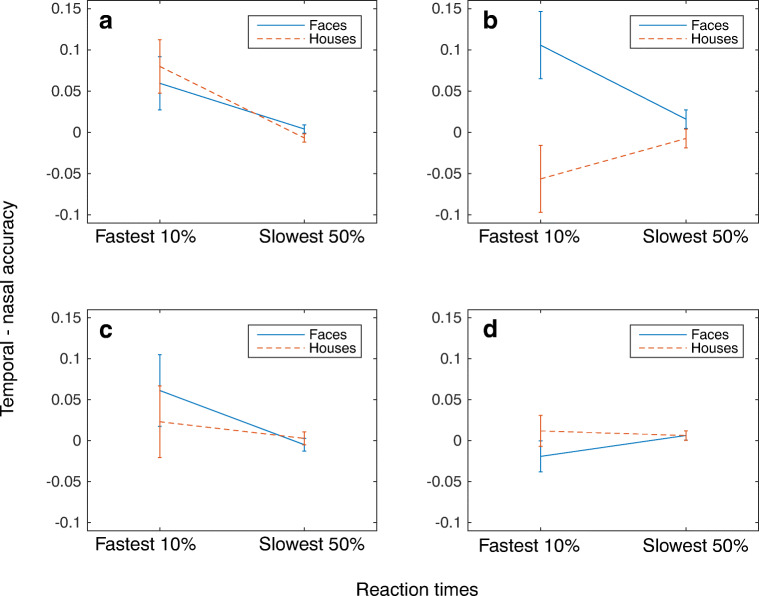
The difference in accuracy between the temporal and nasal hemiretina is plotted for the fastest 10% and slowest 50% reaction times for Experiments 1–4 (**A–D**, respectively). There were no significant differences between the nasal and temporal hemiretina for the faces and houses in any of the experiments. Error bars represent ± 1 standard error

To probe rapid mechanisms, analyses were confined to trials with a rapid response in the fastest 10% of RTs for each category. Participants were able to more accurately detect faces than houses (*F*(1,21)=10.41 *p*<0.01) (Fig. 4 A). This shows that our paradigm is sensitive to rapid, accurate face detection. We then turned to the effect of the retinal hemifield manipulation. There was no overall benefit of presenting stimuli to a particular hemiretina (*F*(1,21)= 3.87, *p*>0.05), suggesting no general role for the retinocollicular pathway in fast visual detection. Furthermore, contrary to what would be expected if the retinocollicular pathway was category selective, and supported rapid face detection, there was no significant stimulus by retinal hemifield interaction (*F*(1,21)=0.1, *p*>0.05) (Fig. 5A). In fact, there was a trend for better performance for faces in the temporal hemiretina.

### Interim discussion

The results of Experiment 1 demonstrate that there is a rapid route for detecting faces that does not extend to other classes of stimuli (i.e., houses). As there was no benefit for presenting stimuli to the nasal hemiretina, the results of the experiment did not provide any evidence of a role for the retinocollicular pathway in rapid visual detection or rapid face processing. The lack of contribution from the retinocollicular pathway, taken with the trend for better processing in the temporal hemiretina, suggests that a cortical route could be responsible for the rapid face detection seen in the experiment.

Our next goal was to probe the specificity of the rapid pathway. A key feature of the rapid route discussed in the literature is that it is not just quick, but that it is dirty (i.e., a coarse representation). In an evolutionary context, it might be advantageous for neural structures to obtain extremely quick, coarse representations of the faces in the environment. This route is not thought to be capable of fine discrimination. Thus, the next experiment was designed to probe the precision of the rapid detection mechanism identified in Experiment 1.

## Experiment 2

### Methods

In order to examine the precision of the rapid cortical detection route, participants performed the same task as in Experiment 1, but with less warped foil stimuli. These foil stimuli still had some recognizable features of faces and houses. If detection relied on a rapid route, exclusively for faces, it would support the idea that the rapid detection mechanism was capable of precise representations. Otherwise, the rapid detection mechanism might be limited to rapid, coarse judgments.

#### Participants

The same participants who participated in Experiment 1 participated in Experiment 2, and the order in which participants completed the two experiments was counterbalanced. Again, two participants were excluded for failing to follow the task instructions.

#### Stimuli and procedure

The stimuli and procedure were the same as in Experiment 1, except that the foil images had less warping applied (image 5 in the diffeomorphic continuum). Examples of the stimuli can be found in Fig. 1B.

### Results

As in Experiment 1, overall mean RTs for the fastest 10% and slowest 50% of trials are shown in Fig. 3B. The mean RTs include both correct responses and false alarms. We used the same analysis procedure as in Experiment 1, with accuracy in the fastest 10% of trials used to assess rapid face detection. When participants were required to make precise judgments, faces were no longer detected reliably more accurately than houses (*F*(1,21)=0.08, *NS*) (Fig. 4B). Again, to examine the role of the retinocollicular pathway we compared presentations to the nasal or the temporal hemiretina. At the fastest RTs, there was no significant difference in accuracy between hemiretinas (*F*(1,21)=0.29, *NS*). Furthermore, again there was no evidence that faces were detected significantly more accurately than houses in the nasal hemiretina when compared to the temporal hemiretina (*F*(1,21)= 3.97, *NS* (Fig. 5B).

In Experiment 1, we found evidence of a fast face-processing mechanism when faces were clearly distinct from foils. In Experiment 2, with a smaller difference between faces and foils, we did not find the same effect. However, it is important to establish whether the effect of the foil manipulation was significant, by directly testing whether the results of the two experiments are significantly different. This comparison showed that performance was significantly more accurate in Experiment 1 than in Experiment 2 (*F*(1,21)=6.81, *p*<0.05). Furthermore, there was a significant interaction between the experiments and stimulus type (*F*(1,21)=8.03, *p*<0.05). This is driven by a greater difference between rapid detection of faces and houses in Experiment 1 than in Experiment 2.

### Interim discussion

When foil stimuli were created with less warping, requiring participants to make fine discriminations, faces were no longer detected more accurately than houses at the fastest RTs. Again, there was not a significant advantage, or a trend for better performance, when stimuli were preferentially presented to the retinocollicular pathway. The results of this experiment support the idea that rapid detection of faces is limited to coarse visual characteristics. When taking Experiment 1 and Experiment 2 together, the results support the idea that there is no advantage of presenting to subcortical structures.

One weakness of the current analysis is that the comparisons of the nasal and temporal hemiretina contain half as much data as the collapsed analyses, and perhaps the consequently reduced power that results is responsible for the lack of significance. Thus, we conducted a further experiment, to double the number of subjects for this comparison. Given recent concerns about the reproducibility of results in psychology (Open Science Foundation, 2015), this also affords us the opportunity to test for replication of the other findings from Experiment 1.

## Experiment 3

### Methods

Experiment 3 was conducted to ensure that the results from Experiment 1 were generalizable, replicating it in a different group of participants. We sought to combine the participants from Experiment 1 and Experiment 3 into a larger analysis, where we would have increased power to detect differences in performance between the nasal and temporal hemiretina.

#### Participants

Twenty-five self-reported right-handed individuals (12 males, 13 females, age range 18–42 years) participated in Experiments 3 and 4. Twenty-four participants reported normal or corrected-to-normal vision. One participant did not have corrected-to-normal vision; their prescription was +0.75 for the right eye and +0.5 for the left eye. Two participants were excluded from the experiment, one because a fire alarm occurred during their experimental session and the other because of technical difficulties that prevented button presses from being recorded.

The participants received $10 for their participation in the experiment. All participants provided written informed consent. The non-medical ethics board at the University of Western Ontario reviewed and approved the experimental protocol.

#### Stimuli and procedure

Stimuli were identical to those that were used in Experiment 1. One important change was made to the procedure. In order to gain information about the participants’ RTs in both warped and intact trials, participants were instructed to press two buttons, one for the warped images and another for the intact images. Exemplar images of the stimuli can be found in Fig. 1A.

### Results

Reaction times for the fastest 10% and slowest 50% of trials are shown in Fig. 3C.

As in Experiments 1 and 2, accuracy in the fastest 10% of trials was examined. In this experiment, we included the data from both the target and the foil trials in our analysis. Replicating the findings from Experiment 1, faces were detected significantly more accurately than houses at faster RTs (*F*(1,22)= 6.24, *p*<0.05) (Fig. 4C). Again, when collapsed across faces and houses, no difference in accuracy at fast RTs was found across the nasal and temporal hemiretina (*F*(1,22)=1.88 *NS*). Furthermore, the interaction between the visual field and stimulus class showed that faces were not significantly more accurate than houses in the nasal hemiretina than the temporal hemiretina (*F*(1,22)=.19, *NS*) (Fig. 5C).

In order to test if a difference in response bias was responsible for the difference in accuracy at the fastest RTs, we also calculated the false-alarm and hit rate for the faces and houses. We were able to do this in Experiment 3 because it was a two-button response task, which allowed us to bin all responses by RT. The mean false-alarm rate was lower for faces (*M*=.21, *SE*=0.016) than for houses (*M*=.25, *SE*=0.016) at the fastest 10% of RTs. The mean hit rate was higher for faces (*M*=.91, *SE*=0.017) than for houses (*M*=.82, *SE*=0.017) at the fastest 10% of RTs. A higher hit rate and a lower false-alarm rate shows the results were not driven by a response bias and participants were actually better at identifying faces than houses. The higher hit rate and low false-alarm rate for faces suggests that participants were not merely responding less carefully to the rapid face trials and that the results were not a result of a speed accuracy trade off.

Although a response bias does not appear to be causing the results in the experiment, it is possible that the effect of hemifield is not being seen because of insufficient power. Therefore, we conducted further analyses in which we included participants from both Experiment 1 and Experiment 3, yielding N=45. When comparing the results of Experiment 1 to Experiment 3, we tested whether the results from the two experiments were significantly different; they were not (*F*(1,43)=1.23, *NS*). In both the nasal and the temporal hemiretina, a significant difference in accuracy at fast RTs was found for face compared with house detection (*t*(1,44)=2.03, *p*<0.05, *t*(1,44)=3.91, *p*<0.001, respectively). This supports the idea that increases in face-detection accuracy are not driven exclusively by an increase in performance in the nasal hemiretina, as would be expected if the retinocollicular pathway were responsible.

Further combined analyses from Experiment 1 and Experiment 3 replicated the key results. At the fastest RTs, faces were detected more accurately than houses (*F*(1,44)=16.44, *p*<0.001), consistent with the results of previous experiments. In addition, at the fastest RTs, overall performance in the nasal hemiretina was significantly worse than performance in the temporal hemiretina (*F*(1,44)=5.74, *p*<0.05). With the larger sample, there was still no significant interaction between stimulus and field (*F*(1,44)=0.03, *NS*) as would be expected if a nasal benefit was driving improved face detection.

One criticism of the approach we have taken is that frequentist statistics only allow for the inability or ability to reject the null hypothesis, whereas Bayesian statistics allow us to estimate the probability of null and other models. In order to address this, in our pooled analysis (45 participants over Experiments 1 and 3), we conducted a Bayesian repeated-measures ANOVA with default prior settings in JASP. There was moderate evidence against a field and stimulus interaction (BF_10_=4.6). A difference would be expected between the nasal faces and houses if the retinocollicular pathway was driving the effects.

### Interim discussion

Experiment 3 replicated the results of Experiment 1, generalizing the findings to a different group of participants and a slightly different response procedure. In addition, calculating the false-alarm and hit rates allowed us to determine that a response bias was not the cause of our results. A higher hit rate and a lower false-alarm rate for faces suggests that increased accuracy is not a result of a speed accuracy trade off. This is further emphasised because faces have a faster mean RT than houses.

Combining the results from Experiments 1 and 3 into a single analysis revealed that presentation to the nasal hemiretina led to significantly worse rapid detection of faces and houses. This result is contrary to what would be expected if the nasal hemiretina, and thus the retinocollicular pathway, were driving the results. In addition, when looking at frequentist statistics, both the nasal and the temporal hemiretina show evidence of significantly more accurate face detection at fast RTs, demonstrating that there is not one hemiretina driving the fast face-detection advantage.

Taken together, these results provide support for the idea that a cortical, rather than a subcortical, process is responsible for rapid face detection. However, we want to be clear that the conclusion is based on our inability to reject the null hypothesis over multiple experiments.

Why might presenting to the nasal hemiretina result in reduced detection of visual stimuli? It is possible that reduced performance could be caused by distracting information (i.e., emotional content) being communicated from subcortical structures to cortical structures. At fast RTs, the brain might be capable of attending only to a subset of information, and emotional content might take precedence over visual categorization, decreasing the accuracy of the nasal hemiretina in Experiments 1 and 3.

In a final experiment we control for a potential low-level visual explanation for the category specificity of the rapid detection mechanism. In our stimulus sets (and more generally, Awasthi, Sowman, Friedman, & Williams, 2013), faces contained lower spatial frequencies than houses. Natural images generally have greater power at lower frequencies (Burton & Moorhead, 1987) so perhaps we have more rapid mechanisms for low spatial frequencies that process faces more rapidly. Thus, perhaps spatial frequency, rather than category *per se*, is responsible for the category-specific rapid detection we observed in Experiments 1 and 3.

## Experiment 4

### Methods

In Experiment 4 we repeated Experiment 3, but the face and house stimulus sets filtered so that they had balanced power spectra.

#### Participants

Experiment 4 tested the same participants as Experiment 3, and the order in which they participated was counterbalanced. Again two participants were excluded – one because of a fire drill and the other because of technical difficulties that prevented button presses from being recorded.

#### Stimuli and procedure

**Fig. 6:**
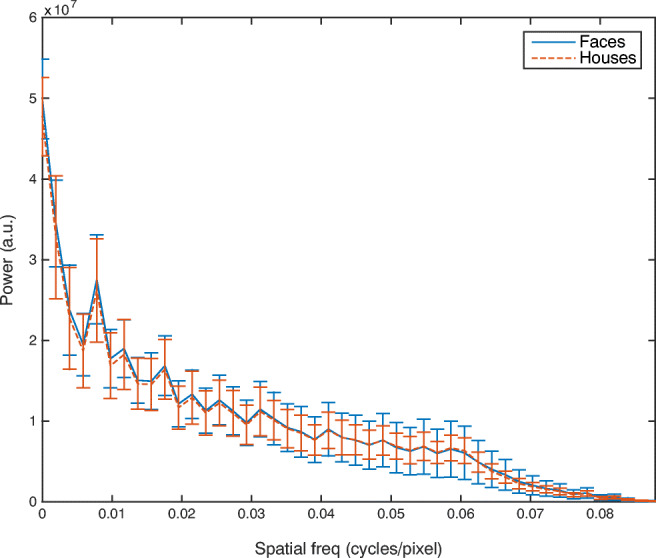
Power of the faces and houses at each spatial frequency (cycles/image)

The same stimuli that were used in Experiments 1 and 3 were used in Experiment 4, but with the spatial frequency of the images balanced. Each image was transformed into 2D frequency space using a Fourier transform. Each pixel was then multiplied by a scalar filtering function that depended only on distance from the origin of frequency space. Finally, an inverse Fourier transform was used to return to image space. Houses were filtered to remove high spatial frequency information, and faces were filtered to remove low spatial frequency information. A further processing stage was applied to remove a visually salient artefact, which was the bleeding of images into the background surrounding them. All voxels outside of each object in the original image (i.e., that were exactly background color) were reset to the background color after filtering. This led to a slight residual mismatch in the resulting frequency spectra, which can be seen in the original and final frequency spectra shown in Fig. 6. Exemplar images can be found in Fig. 1C. All other aspects of the experiment were the same as in Experiment 3.

### Results

Reaction times for the fastest 10% of trials and the slowest 50% of trials can be found in Fig. 3D.

As we obtained data from both target and foil trials, both were included in our analysis. In the fastest 10% of trials, faces were again detected more accurately than houses, despite the matching of spatial frequencies (*F*(1,22)=4.83, *p*<0.05) (Fig. 4D).

Again, the contribution of the retinocollicular pathway was assessed. No significant differences in accuracy were seen for the nasal compared with the temporal hemiretina (*F*(1,22)=0.02, *NS*). The interaction between hemiretina presentation and stimulus was also not significant; houses were not detected significantly more accurately than faces at fast RTs when contrasting the temporal with the nasal hemiretina (*F*(1,22)=.67, *NS*) (Fig. 5D).

To investigate whether spatial frequency manipulation substantially modulated performance, the fastest 10% of trials from Experiment 3 were compared to the fastest 10% of trials from Experiment 4 using a 2 × 2 ANOVA with experiment and stimulus as the within-subject factors. Overall, there were no significant differences in performance between the two experiments *(F*(1,22)=2.80, *p*>0.05). Furthermore, there was no significant interaction between stimulus and experiment, showing the difference in accuracy for faces compared with houses was not significantly different in Experiments 3 and 4 (*F*(1,22)=0.60, *p*>0.05). In line with the results of each experiment, there was a main effect for stimuli, with faces detected significantly more accurately than houses at the fastest RTs (*F*(1,22)=10.17, *p*<0.01).

To further investigate if response bias caused the differences in accuracy at the fastest RTs, we calculated the false-alarm and hit rates for the faces and houses. The mean false-alarm rate was lower for faces (*M*=.13, *SE*=0.018) than for houses (*M*=0.15, *SE*=0.018) at the fastest 10% of RTs. The mean hit rate was higher for faces (*M*=.91, *SE*=0.017) than for houses (*M*=.83, *SE*=0.017) at the fastest 10% of RTs. A higher hit rate and a lower false-alarm rate again confirms that response bias cannot account for the differences in accuracy, and that participants were better at identifying faces than houses.

### Interim discussion

This experiment explored the idea that spatial frequency might have caused the category-specific effects in Experiments 1 and 3. Altering the spatial frequency of the images did not have any significant effect on the results. In trials with fast RTs, faces were still detected more accurately than houses. Furthermore, the spatial filtering in Experiment 4 did not change the results from those seen in Experiment 3. These results support a face-specific rapid detection mechanism, rather than a low-spatial frequency mechanism.

## General discussion

Four experiments were conducted to determine whether a rapid route for face detection could be identified in a behavioral experiment. In addition, we sought to determine whether a subcortical process, facilitated by the retinocollicular pathway, could be responsible for the rapid detection of faces. If the retinocollicular pathway to the amygdala was responsible for rapid face detection, we would expect to see a benefit for faces, but not houses, when presenting to the nasal hemiretina. In Experiments 1, 3, and 4, participants rapidly detected faces but not houses from very distinct warped foil stimuli. However, there was no benefit of presenting the stimuli to the nasal hemiretina, providing no support for a retinocollicular route in rapid face detection in our task. Even when we combined the participants from Experiments 1 and 3 into a single analysis to increase power, we did not see a benefit for face detection in the nasal hemiretina, and in fact, faces or houses presented to the nasal hemiretina were detected less accurately.

We then considered what aspects of the face stimuli could have led to rapid detection. Faces have greater power at lower spatial frequencies than houses. In Experiment 4, we filtered the images to enhance relative power at high spatial frequencies for the faces and reduce it for the houses. Faces were still detected more accurately than houses, showing that it is category, and not just spatial frequency, that facilitates rapid detection. Furthermore, we found performance overall was no worse when high frequencies were emphasized. This suggests low spatial frequencies did not have a strong role, and that perhaps the rapid detection mechanism is capable of precise visual representation. We tested this in Experiment 2 and found that when participants were required to make fine visual discriminations, more accurate fast face detection disappeared. This suggests the rapid discrimination method is “dirty” as well as being “quick.” Again, no contribution was evident from the retinocollicular pathway.

Taken together, our results show there is a rapid route for the detection of faces, which relies on coarse visual information, but not low on spatial frequencies in particular. In none of the experiments did we find evidence of a benefit for face detection in the nasal hemiretina. This could support the idea that a cortical rather than a subcortical mechanism is responsible for rapid face detection (Cauchoix, & Crouzet, 2013), and is congruent with evidence that the cortex is capable of rapid processing (Barragan-jason et al., 2015; Foxe & Simpson, 2002; H. Liu et al., 2009). However, we acknowledge that no imaging (e.g., fMRI) was performed in this study. Therefore, although our behavioral experiment may motivate future imaging work, it is not possible for us to determine the brain structures responsible for the behavior in these experiments.

Strengthening the results of the study, the warped foil stimuli used in this experiment were well matched in terms of luminance, contrast, and spatial frequency to the target stimuli, and could not be differentiated in a model of the early visual system

(HMAX; Stojanoski & Cusack, 2014), eliminating a series of confounding variables not often considered. There is one study where the authors found participants were orienting more quickly to “face-like” stimuli when they were presented to the nasal hemiretina (Tomalski, Johnson, & Csibra, 2009). However, this study used “Johnson faces” where black boxes are put in place of the eyes, nose, and mouth. Control stimuli in this study were an inverted version of the “Johnson face.” Although these control stimuli were matched for variables like spatial frequency, the target stimuli will have a large “top-heavy” bias in comparison with the foil stimuli, which could be what was responsible for the increased performance of the nasal hemiretina. Our naturalistic stimuli will likely have had less of a top-heavy bias, and this could be a potential reason why we do not see a benefit of presenting to the nasal hemiretina. It is also possible that the cortex is needed to make category judgments when target and foil stimuli are well matched.

In order to ensure that the visual stimuli were unrecognizable, Stojanoski and Cusack (2014) quantified how much warping was necessary to remove semantic information from different categories. Faces, along with bikes, needed the highest levels of warping in order to render them unrecognizable. Therefore, it is unlikely that face blocks in Experiments 1 and 3 represented an easier task than house blocks. In addition, different diffeomorphic fields were used for each foil, which makes them distinct, even within a category. For example, although the first exemplar in Fig. 1 has a dark portion in the center, not all face stimuli have this. Across the entire face and house categories, these small characteristics were insufficient to have driven the broader differences between the faces and the houses.

Other researchers have also failed to see a benefit for face identification when presenting stimuli to the nasal hemiretina (Gabay, Burlingham, & Behrmann, 2014). In addition to the nasal/temporal manipulation, these researchers use a Wheatstone stereoscope to exploit the fact that visual information is segregated monocularly until the visual cortex. Gabay et al. (2014) presented stimuli monocularly, either to the same or different eyes, and had participants make identity judgements. They found a benefit for presenting stimuli to the same eye, which they hypothesize could be due to the monocular properties of subcortical structures, such as the lateral geniculate nucleus (LGN). Although the retinocollicular pathway does not seem to be contributing to improved face detection, it is possible that the LGN, on the way to the cortex, could be responsible for our results.

If the amygdala is not responsible to the rapid detection of faces, it could still be processing emotional information (Vuilleumier, Richardson, Armony, Driver, & Dolan, 2004). This could explain why we see decreased overall performance for the nasal hemiretina. It is possible that when the amygdala feeds information to the cortex creating competing processing, it makes more difficult for the cortex to rapidly categorize visual stimuli.

If faces are being detected more accurately at fast RTs than houses, what features of the stimuli are causing this increase in accuracy? Faces have significantly less inter-exemplar variability than houses. It is possible that the invariance of face stimuli allows tighter tuning in cortex, leading to more accurate, robust, and efficient detection. If the invariability in our stimuli is causing the effects seen in the experiments, it is possible that other stimulus categories with limited variability could tap into a rapid mechanism. If other categories of stimuli could be capable of tapping into the rapid mechanism, are faces really special or is expertise what is important in order to develop “expert” face-processing capabilities? Several studies have highlighted how important experience is in the processing of faces. For example, cataracts that substantially decrease visual input from reaching the right hemisphere in infancy impair “expert” face processing from completely developing (Le Grand, Mondloch, Maurer, & Brent, 2003). There is also evidence that perceptual narrowing and other complex aspects of face processing continue to emerge over the first year of life, substantiating the hypothesis that experience is important in face processing (Kelley, Quinn, Slater, Lee, Ge, & Pascalis, 2008; Sai, 2005). However, other researchers have found that the cortex responds to faces extremely quickly after birth (Tzourio-Mazoyer et al., 2002), and dispute the experience hypothesis (McKone, Crookes, Jeffery, & Dilks, 2012). Our results could suggest that other categories of stimuli with limited variability and increased experience could tap into this rapid route.

Another theoretical framework that our results could be considered in is the dual-process theory. Proponents of dual-process theories have suggested that there are two processes involved in cognition, the first an unconscious process (often thought of as procedural learning), and the second a conscious, effortful process (i.e., explicit learning) (Barrett, Tugade, & Engle, 2004). In the categorization literature, others have proposed a dual process model specific to categorization (COVIS), which has a procedural learning component and a cognitively demanding, verbal hypothesis-driven component, mediated by the executive network (Ashby, Alfonso-Reese, Turken, & Waldron, 1998; Maddox & Ing, 2005). It could be that faces access a rapid procedural mechanism while slower categorization is dominated by the explicit process. However, other researchers have criticized COVIS (Newell, Dunn, & Kalish, 2011). From our current data is impossible to determine whether our results are reflective of a dual process theory or are the result of a single process that is more robust to faces. Future work should seek to examine this.

## Conclusions

In conclusion, faces were detected with greater accuracy at fast RTs than houses, when they are distinct from the foil stimuli. Our data do not offer any support that these results are due to the contributions of the retinocollicular pathway, suggesting that an alternative route to cortex is involved in the rapid detection of faces.

## Electronic supplementary material

ESM 1 (DOCX 62 kb)

## References

[CR1] Ashby FG, Alfonso-Reese LA, Turken AU, Waldron EM (1998). A neuropsychological theory of multiple systems in category learning. Psychological Review.

[CR2] Awasthi B, Sowman PF, Friedman J, Williams MA (2013). Distinct spatial scale sensitivities for early categorization of faces and places: neuromagnetic and behavioral findings. Frontiers in Human Neuroscience.

[CR3] Barragan-jason G, Cauchoix M, Barbeau EJ (2015). The neural speed of familiar face recognition. Neuropsychologia.

[CR4] Barrett LF, Tugade MM, Engle RW (2004). Individual Differences in Working Memory Capacity and Dual-Process Theories of the Mind. Psychological Bulletin.

[CR5] Benevento LA, Standage GP (1983). The organization of projections of the retinorecipient and nonretinorecipient nuclei of the pretectal complex and layers of the superior colliculus to the lateral pulvinar and medial pulvinar in the macaque monkey. The Journal of Comparative Neurology.

[CR6] Bertini C, Leo F, Làdavas E (2008). Temporo-nasal asymmetry in multisensory integration mediated by the Superior Colliculus. Brain Research.

[CR7] Burton GJ, Moorhead IR (1987). Color and spatial structure in natural scenes. Applied Optics.

[CR8] Cauchoix S, Crouzet MC (2013). How plausible is a subcortical account of rapid visual recognition ?. Frontiers in Human Neuroscience.

[CR9] Celebrini S, Thorpe S, Trotter Y, Imbert M (1993). Dynamics of orientation coding in area V1 of the awake primate. Visual Neuroscience.

[CR10] Conley, M., Lachica, E. A., & Casagrande, V. A. (1985). Demonstration of ipsilateral retinocollicular projections in the tree shrew (Tupaia glis). *Brain Research* (Vol. 346). 10.1016/0006-8993(85)91113-8 4052767

[CR11] Crouzet SM, Kirchner H, Thorpe SJ (2010). Fast saccades toward faces: face detection in just 100 ms. Journal of Vision.

[CR12] Delorme A, Thorpe SJ (2001). Face identification using one spike per neuron: Resistance to image degradations. Neural Networks.

[CR13] Fletcher-Watson S, Findlay JM, Leekam SR, Benson V (2008). Rapid detection of person information in a naturalistic scene. Perception.

[CR14] Foundation, O. S. (2015). Estimating the reproducibility of psychological science. *Science*, *349*(6251). doi:10.1126/science.aac4716 10.1126/science.aac4716 26315443

[CR15] Foxe, J. J., & Simpson, G. V. (2002). Flow of activation from V1 to frontal cortex in humans A framework for defining “ early ” visual processing 139–150. doi:10.1007/s00221-001-0906-7 10.1007/s00221-001-0906-7 11797091

[CR16] Gabay S, Burlingham C, Behrmann M (2014). The nature of face representations in subcortical regions. Neuropsychologia.

[CR17] Garvert MM, Friston KJ, Dolan RJ, Garrido MI (2014). Subcortical amygdala pathways enable rapid face processing. NeuroImage.

[CR18] Harrison DW (2015). *Brain Asymmetry and Neural Systems*.

[CR19] Haxby J, Hoffman E, Gobbini M (2000). The distributed human neural system for face perception. Trends in Cognitive Sciences.

[CR20] Johnson MH (2005). Subcortical face processing. Nature Reviews. Neuroscience.

[CR21] Jolij J, Lamme VAF (2005). Repression of unconscious information by conscious processing: Evidence from affective blindsight induced by transcranial magnetic stimulation. Proceedings of the National Academy of Sciences.

[CR22] Jones, E. G., & Burton, H. (1976). A projection from the medial pulvinar to the amygdala in primates. *Brain Research* (Vol. 104). 10.1016/0006-8993(76)90654-5 813820

[CR23] Kanwisher N, Mcdermott J, Chun MM (1997). The Fusiform Face Area: A Module in Human Extrastriate Cortex Specialized for Face Perception. The Journal of Neuroscience.

[CR24] Kanwisher, N., & Yovel, G. (2006). The fusiform face area: a cortical region specialized for the perception of faces, (November), 2109–2128. doi:10.1098/rstb.2006.1934 10.1098/rstb.2006.1934 PMC1857737 17118927

[CR25] Kelley D, Quinn P, Slater A, Lee K, Ge L, Pascalis O (2008). The Other-Race Effect Develops During Infancy. Psychological Science.

[CR26] Kirchner H, Thorpe SJ (2006). Ultra-rapid object detection with saccadic eye movements: Visual processing speed revisited. Vision Research.

[CR27] Konig, P., Engel, A. K., Roelfsema, P. R., & Singer, W. (1995). How Precise is Neuronal Synchronization? 10.1162/neco.1995.7.3.469 8935960

[CR28] Le Grand R, Mondloch CJ, Maurer D, Brent HP (2003). Expert face processing requires visual input to the right hemisphere during infancy. Nature Neuroscience.

[CR29] Leopold DA, Rhodes G (2010). NIH Public Access.

[CR30] Liu H, Agam Y, Madsen JR, Kreiman G (2009). Article Timing, Timing, Timing: Fast Decoding of Object Information from Intracranial Field Potentials in Human Visual Cortex. Neuron.

[CR31] Liu J, Harris A, Kanwisher N (2002). Stages of processing in face perception: an MEG study. Nature Neuroscience.

[CR32] Luo Q, Holroyd T, Majestic C, Cheng X, Schechter J, Blair RJ (2010). Emotional Automaticity Is a Matter of Timing. Journal of Neuroscience.

[CR33] Maddox TW, Ing DA (2005). Delayed Feedback Disrupts the Procedural-Learning System but Not the Hypothesis-Testing System in Perceptual Category Learning. Journal of Experimental Psychology.

[CR34] Martin CB, McLean DA, O’Neil EB, Köhler S (2013). Distinct familiarity-based response patterns for faces and buildings in perirhinal and parahippocampal cortex. The Journal of Neuroscience: The Official Journal of the Society for Neuroscience.

[CR35] McKone E, Crookes K, Jeffery L, Dilks DD (2012). A critical review of the development of face recognition: experience is less important than previously believed. Cognitive Neuropsychology.

[CR36] Mende-Siedlecki, P., & Verosky, S. (2013). Robust selectivity for faces in the human amygdala in the absence of expressions, 2086–2106. doi:10.1162/jocn 10.1162/jocn_a_00469 PMC4029947 23984945

[CR37] Méndez-Bértolo C, Moratti S, Toledano R, Lopez-Sosa F, Martínez-Alvarez R, Mah YH (2016). A fast pathway for fear in human amygdala. Nature Neuroscience.

[CR38] Newell, B. R., Dunn, J. C., & Kalish, M. (2011). Systems of Category Learning: Fact or Fantasy? In *The Psychology of Learning and Motivation* (Vol. 54, pp. 167–215). Academic Press. doi:10.1016/B978-0-12-385527-5.00006-1

[CR39] Perry VH, Cowey A (1985). The ganglion cell and cone distributions in the monkey’s retina: implications for central magnification factors. Vision Research.

[CR40] Pitcher D, Dilks DD, Saxe RR, Triantafyllou C, Kanwisher N (2011). Differential selectivity for dynamic versus static information in face-selective cortical regions. NeuroImage.

[CR41] Pitcher D, Walsh V, Yovel G, Aviv T (2007). TMS Evidence for the Involvement of the Right Occipital Face Area in Early Face Processing. Current Biology.

[CR42] Pollack JG, Hickey TL (1979). The distribution of retino-collicular axon terminals in rhesus monkey. The Journal of Comparative Neurology.

[CR43] Rafal RD, Koller K, Bultitude JH, Mullins P, Ward R, Mitchell AS, Bell AH (2015). Connectivity between the superior colliculus and the amygdala in humans and macaque monkeys: virtual dissection with probabilistic DTI tractography. Journal of Neurophysiology.

[CR44] Sadeh B, Podlipsky I, Zhdanov A, Yovel G (2010). Event-related potential and functional MRI measures of face-selectivity are highly correlated: A simultaneous ERP-fMRI investigation. Human Brain Mapping.

[CR45] Sai, F. Z. (2005). The Role of the Mother ’ s Voice in Developing Mother’s Face Preference: Evidence for Intermodal Perception at Birth, *50*, 29–50. doi:10.1002/icd.

[CR46] Sato W, Kochiyama T, Uono S, Matsuda K, Usui K, Inoue Y, Toichi M (2013). Rapid and multiple-stage activation of the human amygdala for processing facial signals. Communicative & Integrative Biology.

[CR47] Serre, T., Oliva, A., & Poggio, T. (2007). A feedforward architecture accounts for rapid categorization. *Proceedings of the National Academy of Sciences*, *104*. 10.1073/pnas.0700622104 PMC1847457 17404214

[CR48] Sterling P (1973). Quantitative mapping with the electron microscope: retinal terminals in the superior colliculus. Brain Research.

[CR49] Stojanoski, B., Cusack, R. (2014). Time to wave good-bye to phase scrambling: Creating controlled scrambled images using diffeomorphic transformations, *14*, 1–16. doi:10.1167/14.12.6.doi 10.1167/14.12.6 25301014

[CR50] Sylvester, R., Josephs, O., Driver, J., & Rees, G. (2007). Visual fMRI Responses in Human Superior Colliculus Show a Temporal – Nasal Asymmetry That Is Absent in Lateral Geniculate and Visual Cortex, (Fries 1984), 1495–1502. doi:10.1152/jn.00835.2006. 10.1152/jn.00835.2006 17135475

[CR51] Tamietto M, de Gelder B (2010). Neural bases of the non-conscious perception of emotional signals. Nature Reviews. Neuroscience.

[CR52] Tamietto M, Pullens P, De Gelder B, Weiskrantz L, Goebel R (2012). Subcortical connections to human amygdala and changes following destruction of the visual cortex. Current Biology.

[CR53] Tomalski P, Johnson MH, Csibra G (2009). Temporal-nasal asymmetry of rapid orienting to face-like stimuli. Neuroreport.

[CR54] Tong F, Nakayama K, Moscovitch M, Weinrib O, Kanwisher N (2000). Response properties of the human fusiform face area. Cognitive Neuropsychology.

[CR55] Tzourio-Mazoyer N, De Schonen S, Crivello F, Reutter B, Aujard Y, Mazoyer B (2002). Neural correlates of woman face processing by 2-month-old infants. NeuroImage.

[CR56] VanRullen R, Guyonneau R, Thorpe SJ (2005). Spike times make sense. Trends in Neurosciences.

[CR57] Vanrullen R, Koch C (2001). Visual Selective Behavior Can Be Triggered by a Feed-Forward Process. Journal of Cognitive Neuroscience.

[CR58] Visconti di Oleggio Castello M, Gobbini MI (2015). Familiar Face Detection in 180 ms. PloS One.

[CR59] Vuilleumier P, Armony JL, Driver J, Dolan RJ (2003). Distinct spatial frequency sensitivities for processing faces and emotional expressions. Nature Neuroscience.

[CR60] Vuilleumier, P., Richardson, M. P., Armony, J. L., Driver, J., & Dolan, R. J. (2004). Distant influences of amygdala lesion on visual cortical activation during emotional face processing, *7*(11), 1271–1278. doi:10.1038/nn1341 10.1038/nn1341 15494727

