# Supplementary material for: Rapid and coarse face detection: With a lack of evidence for a nasal-temporal asymmetry
Source: Atten Percept Psychophys. 2020 Jan 6;82(4):1883–95. doi: 10.3758/s13414-019-01877-3 (PMC7297860; doi:10.3758/s13414-019-01877-3)
Supplement: Supplementary file 1 — (DOCX 62 kb) [file 13414_2019_1877_MOESM1_ESM.docx]

Supplementary Information

Further analysis was done regarding the images used in Experiment 1 and 3.

In order to see how greyscale brightness could have impacted the experiments, we plotted

it against the cumulative portion of pixels. Stimuli were well matched to their warped

counterparts that participants had to differentiate between in each block,

making it unlikely that image brightness contributed to the experimental results.
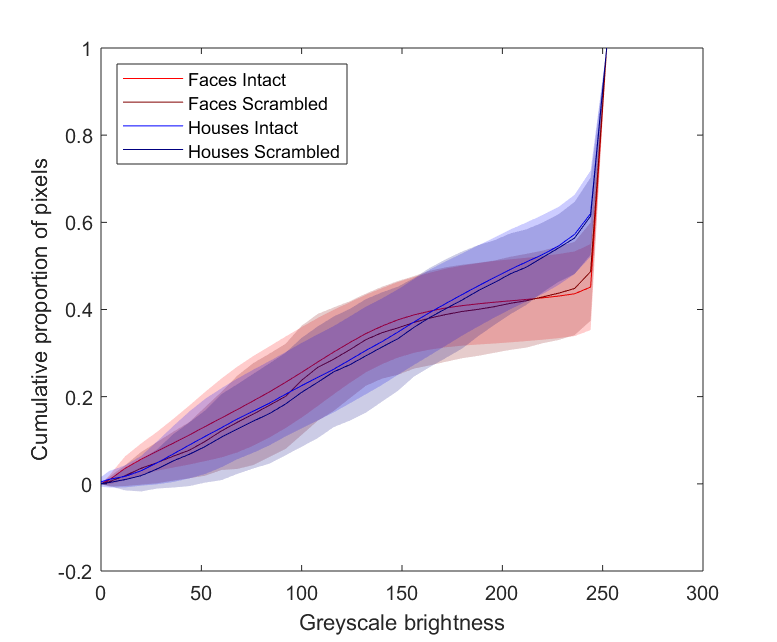


*Supplementary Figure 1.* The number of pixels for cumulative greyscale brightness in each category. The shaded coloring represents +/- 1 standard deviation across images. The warped stimuli were well matched to the intact stimuli that participants had to discriminate between in each block, during Experiments 1 and 3.
